# Gold standard research—reflections on the NIH announcement

**DOI:** 10.1093/haschl/qxaf191

**Published:** 2025-10-07

**Authors:** Howard Bauchner, Frederick P Rivara

**Affiliations:** Department of Pediatrics, Boston University Chobanian and Avedisian School of Medicine, Boston, MA 02118, United States; National University of Singapore, Singapore, Singapore; Department of Pediatrics, University of Washington, Seattle, WA 98195, United States

**Keywords:** research integrity, scientific integrity, ethics

## Abstract

The National Institutes of Health (NIH) recently released a document titled “Leading in Gold Standard Science—An NIH Implementation Plan.” We offer reflections on 4 of the 9 “tenets” of gold standard research and recommendations for improving scholarly publication.

In August, 2025, the National Institutes of Health (NIH) released a document titled “Leading in Gold Standard Science.”^1^ It describes 9 “tenets” of gold standard research: Reproducible, Transparent, Communicative of Error and Uncertainty, Collaborative and Interdisciplinary, Skeptical of Its Findings and Assumptions, Structured for Falsifiability of Hypotheses, Subject to Unbiased Peer Review, Accepting of Negative Results as Positive Outcomes, and Without Conflicts of Interest. It acknowledges numerous initiatives that are already in place at the NIH and indicates that more are planned. In this Commentary we reflect on some of these initiatives, drawing on our experiences as investigators and editors.

## Reproducibility and replication

The document recognizes that reproducible and replicable research are not the same. It defines reproducibility as “the ability of independent researchers to test a hypothesis through multiple methods and consistently achieve results that confirm or refute it, ensuring findings are generalizable and robust across different approaches.” In contrast replicability is “the ability to perform the same experiment or study using the same methods and conditions to achieve the same result.” Both require funding, and reproducibility also requires that data be freely available to both analyze and reanalyze. How individuals doing this work will be recognized, receive academic credit, and be supported requires further discussion.

## Peer review

Artificial intelligence (AI) needs to play some role in the future of peer review.^2^ Current peer review, particularly of manuscripts submitted for publication, can be inefficient, ineffective, and biased. Although there is renewed interest in paying peer reviewers, the number of manuscripts being submitted for publication—likely more than 3 million annually in the biological sciences—precludes any likelihood that publishers will commit to such payment, although the NIH recently requested comment on a proposal to pay peer reviewers as costs included in author processing fees.^3^ In addition, the data regarding outcomes in paying peer reviewers are limited and inconsistent. In a recent trial in critical care medicine, paying peer reviewers $250 reduced the time to return of the review (11 vs 12 days) but had no effect on quality.^4^ There are data suggesting that AI is effective; however, whether it is more effective than human peer review, or less biased, or can provide data protection, is not clear.^5^ But we believe those data will emerge, and that AI combined with human review will be found to be effective, efficient, and less prone to bias than the current approach.

## Transparency

The NIH has tried to ensure greater transparency of its research. For example, the NIH requires that results of randomized clinical trials (RCTs) it supports be posted on clinicaltrials.gov and, more recently, data that underpin NIH studies be made publicly available. Obviously, the key issue will be enforcement, which has been a challenge in the past for NIH data-sharing policies. Interestingly, the Food and Drug Administration (FDA), which is part of the Department of Health and Human Services, has a treasure trove of data—for every drug and device they approve they have detailed information on every single patient enrolled in those studies. Given the importance of drug and device approval, it is unfortunate those data are not made publicly available. The FDA appears to be making a commitment to greater transparency, as the release of Complete Response Letters highlights.^6^

## Conflicts of interest

Conflicts of interest certainly can be about money but often go far beyond the almighty dollar. Expert panels, such as the Advisory Committee on Immunization Practices (ACIP) and the US Preventive Services Task Force (USPSTF), have been traditionally assembled based on the premise that experts in their respective fields will make the best possible decisions based upon the science available to them. The dissolution of the ACIP was ostensibly done because of financial conflicts of interest (COIs), which is not true.^7^ Some of the nominated replacements for ACIP have a different type of COI—strongly held beliefs.

In this dueling discussion of COI—financial vs cognitive—which group is more at risk of having their COI affect policy recommendations? Is the original panel of ACIP members without COIs? Assuming they do not have financial COIs, they clearly have other COIs. Anyone who spends their career studying a topic and developing deep expertise in it cannot be said to have no COIs. Their COIs may be in the belief that gold standard science as suggested by the NIH may be the best way to make a valid recommendation to optimize the health of the public.

These same COIs may also affect peer review, whether it be of grant applications or journal manuscripts. Many of us have been the recipient of reviews that we felt were not only inaccurate but biased, reflecting that reviewers brought their years of experience, theoretical framework, and disciplinary background to the review.

What is the way forward? How do we manage these COIs to support gold standard science that fosters the health of the public, the funding of a grant, or the publication of a manuscript? Financial COIs can be managed; cognitive bias is more challenging. For manuscripts and grants, AI may help. For panels such as ACIP or the USPSTF, beyond ensuring that individuals have sufficient expertise, those with strongly held immutable beliefs, often reflected in manuscripts, social media, or podcasts, perhaps should not be members.

## Conclusion

The road to gold standard research is paved with many challenges. Many individuals and groups have been discussing concerns about the integrity of science for decades. However, this has come into sharper focus in recent years because of concerns that there has been an increase in the number of manuscripts retracted, a sense that the public is uncertain about the integrity of science, and an increase in misinformation and disinformation.

With massive amounts of data now available, from large cohorts, electronic health records, and open science, how this will impact the scientific literature is unclear, although it has already led to an increase in the number of published manuscripts, with evidence of data dredging and poorly planned analyses.^8,9^ This will make pursuing gold standard research only more difficult.

There are several recommendations that can improve the quality of science. First, all clinical studies, including observational studies, should be registered prior to data collection. Like with RCTs, investigators should create both a protocol and statistical analytic plan, thus limiting data dredging. This should make reproducibility possible. Second, for studies for which there is broad agreement that registration is necessary (eg, RCTs and meta-analyses) journals should refuse to publish those that are not registered. Third, the NIH and other funders of biomedical research should enforce existing policies, such as access to data, that will improve scientific integrity. Fourth, the FDA should begin to make all data that underpin the approval of drugs and devices publicly available. At the same time they must protect intellectual property. Fifth, journals should move to the adoption of AI to assist editors and peer reviewers in the review of manuscripts. AI is likely to be efficient in ensuring that studies adhere to the many reporting guidelines. Sixth, we acknowledge the importance of hypothesis-generating studies; indeed, they are part of the scientific process. Those manuscripts should be clearly labeled as such to help ensure appropriate interpretation. This would be consistent with several tenets in the NIH statement.

A renewed commitment by the NIH to gold standard research is welcome. As the leading funder of biomedical research in the world, its policies and procedures are influential. It is the responsibility of funding agencies, including foundations, professional societies, and federal agencies, and mentors, investigators, and editors to ensure integrity in science.

## Supplementary Material

qxaf191_Supplementary_Data
